# What role does metal allergy sensitization play in total knee arthroplasty revision?

**DOI:** 10.1186/s40634-018-0146-4

**Published:** 2018-08-14

**Authors:** David R. Lionberger, Justin Samorajski, Charlie D. Wilson, Andreana Rivera

**Affiliations:** 1 Southwest Orthopedic Group LLC, 6560 Fannin Street, Suite 1016, Scurlock Tower, Houston, TX 77030 USA; 2 Texas A&M College of Medicine, Houston Methodist Hospital, 6565 Fannin Street, Houston, TX 77030 USA; 3 Scott & White Memorial Hospital, 2401 S. 31st St, Temple, TX 76508 USA; 4 Department of Pathology, Houston Methodist Hospital, 6565 Fannin Street, Suite M227, Houston, TX 77030 USA

**Keywords:** Metal allergy, Wear byproducts, Hypersensitivity, Ceramic implant, Failed knee arthroplasty

## Abstract

**Background:**

Clinicians are often faced with the decision whether to revise a painful total knee replacement in patients who have chronic vague pain with no apparent explanation. A sensitive metal testing assay called the lymphocyte stimulation test has been used to detect nickel sensitization in patients with orthopedic implants. We hypothesize that nickel sensitization plays a role in the pathology of failed joint arthroplasty in patients with unexplained dissatisfaction.

**Methods:**

32 patients with symptomatic total knee arthroplasty without obvious mechanical findings were tested prior to revision surgery. 19 nickel-sensitized and 13 non-sensitized patients were compared by cell counts of synovium surgical specimens for CD4^+^ and CD8^+^ cell lines. Patients were then revised with ceramic-coated implants. Secondary evaluation of functional outcomes, range of motion, and pain relief were assessed.

**Results:**

Nickel-sensitive patients showed a statistical increase in CD4^+^ reactivity compared to CD8^+^ reactivity. The ratio of CD4^+^/CD8^+^ T lymphocytes was 1.28 in nickel-sensitive patients versus 0.76 in the control (*p* = 0.009). There was no difference in functional scores, clinical scores, or range of motion after revision.

**Conclusions:**

This study provides objective data via histological analysis in support of a nickel allergic sensitization in failed arthroplasties where clinical and/or radiographic abnormalities may not be apparent. Biopsy for CD4^+^/CD8^+^ cell counts may provide further proof of the existence of nickel sensitization in lymphocyte stimulation test positive patients, and more importantly, may implore the surgeon to consider low nickel implant design in these patients.

## Background

Allergic reaction to orthopedic implant metals has not received much attention in the past largely due to the difficulty to test and quantify allergic responses. However, in the last decade, orthopedic surgery has wrestled with metal-wear complications, one of which is the metal-on-metal (MOM) total hip replacement. Until the findings of MOM sensitivity became apparent, allergic sensitization was not perceived as an area of concern (Beecker et al. 2009; Handa et al. 2003; Repantis et al. 2013; Watters et al. 2010). With the popularity of MOM hips reaching 35% of total hip replacements performed, there has been a plethora of articles focused on metal abrasion, corrosion, and resultant tissue response around implants (Grupp et al. 2009; Kwon et al. 2009; Sicilia et al. 2008; Willert et al. 2005). While corrosion may not create as much local tissue hypertrophy, the seemingly asymptomatic MOM hip can cause reactions around the implant, which manifests as pseudo tumor or an aseptic lymphocyte-dominated vasculitis-associated lesion (ALVAL). This ALVAL reaction has become more commonly known as the MOM hypersensitization, which may have created tissue hyperplasia in MOM total hips (Bisschop et al. 2013; Demehri et al. 2014; Gao et al. 2011; Hart et al. 2012; Jacobs and Hallab 2006; Latteier et al. 2011; Schafer et al. 2001; Thomas et al. 2006). A mechanism behind the formation of this tissue injury appears to be metal byproducts of wear such as ionized forms of chromium and nickel which stimulate T-cell sensitization (Caicedo et al. 2013; Ikarashi et al. 2002; Lohmann et al. 2013).

Serum metal ion levels may be elevated in normally functioning implants and ion levels do not accurately predict outcomes of patient satisfaction or implant function (Lachiewicz et al. 2016; Luetzner et al. 2007). While metal ion levels are not a reliable predictor of potential implant failure, a patient’s metal sensitivity status may be a factor in implant failure. The incidence of nickel (Ni) and chromium (Cr) sensitivity in the normal population is approximately 10% (Hallab et al. 2001; Reich et al. 2010). If one has an implant in place, metal exposure may cause the incidence of sensitization to rise as high as 25% (Bloemke and Clarke 2015; Hallab et al. 2001). If the implant mechanically fails, because of loosening or other causes, the incidence can be as high as 60% (Hallab et al. 2001). Testimony to the existence of implant allergy exists not only in skin testing, but also in the histologic testing of T-cell reactivity. Rather than dermatological testing, a more direct and objective T-cell reactivity assay, better known as lymphocyte transformation test (LTT) is used to evaluate for an allergic response. This assay tests for proliferation of peripheral blood lymphocytes in response to a specific immunological challenge such as nickel, cobalt, chromium, iron, or other trace elements (Niki et al. 2005). Proliferation of lymphocytes in response to one of these potential allergens is indicative of a delayed-type (type IV) allergic response. While dermatologic testing may be simpler to perform, the advantage of the LTT is the reduction of false positives and enhanced specificity—especially when testing for metal byproducts of wear—as compared to skin testing (Carando et al. 1985). It avoids confounding variation of metal concentrations used in the dermatologic contact tests and eliminates the subjectivity of skin test interpretation by shifting to an objective numerical value on an assay (Mihalko et al. 2012).

The histological response to a metal allergen is best described as a T-cell-mediated type of hypersensitivity, characterized by activation of both CD4^+^ and CD8^+^ T-cells (Cavani et al. 2003). Delayed-type hypersensitivity reactions to poison ivy and autoimmune disease are well described in the literature (Usatine and Riojas 2010). However, type IV hypersensitivity in orthopedic implants is less understood (Hallab et al. 2001). This activation process initiates when metal ions behave as haptens, activating CD4^+^ cells. Activated CD4^+^ cells produce cytokines such IFN-gamma, TNF-alpha, IL-17, IL-22, and ultimately IL-12 (Hallab et al. 2001; Kumar et al. 2010; Lachiewicz et al. 2016). The resulting activation of the Th_17_ cell line by these cytokines can then induce an inflammatory response that may persist for a long period of time, thus creating the memory effect of what is classically seen in simple skin reactions. This activation cascade may serve as the mechanism of cellular reactivity to metal byproducts that was previously thought unlikely.

This study specifically focuses on metal allergy in patients who have undergone total knee arthroplasty (TKA). When assessing the painful TKA, there are often patients without mechanical or functional findings and often-vague pain for which no apparent explanations for their pain exist (Caicedo et al. 2014). A delayed-type hypersensitivity reaction to metal orthopedic implants is a plausible factor that may contribute to some of these failures (Bergschmidt et al. 2012; Schroer et al. 2013). If hypersensitivity reactions to specific metals like nickel do indeed contribute to TKA failure, one should see a histological response of delayed-type hypersensitivity reactions in failed knees. In this study, we sought to assess the presence of immunologic CD4^+^ and CD8^+^ staining in sections taken from a sequential series of revision TKA patients who were revised for implant failure unrelated to infection or mechanical malfunction and all of whom had undergone lymphocyte transformation testing (LTT) for sensitivity to nickel. We then evaluated the histologic response differences between those sensitive to nickel and a non sensitive control group. The histological findings were also compared to functional and clinical Knee Society scores assessed before and after surgical revision.

## Methods

### Allergy testing

This study was approved by the Houston Methodist Institutional Review Board (No. 12712) and was conducted in accordance with the Declaration of Helsinki. All participants in the study provided written informed consent, testing was self funded by patients. Lymphocyte transformation test (Orthopedic Analysis LLC, Chicago IL) was used to determine if patients were hypersensitive to nickel. The proliferation index is the main result of this assay, it is the ratio of proliferation of lymphocytes exposed to a metal antigen to those exposed to a negative control, measured by radiation counts per minute. A proliferation index of less than 4 is considered no to low reactivity, while greater than 4 is considered reactive to highly reactive, defined as a statistically significant > 4 fold proliferation index response, *p* < 0.05). The patients who had a LTT < 4 were classified as controls and patients who had a LTT > 4 were classified as nickel sensitive. One week prior to testing, the patients were taken off immunologic-suppressive agents including NSAIDs, which could compromise the sensitivity of the test.

### Patient selection

The sequential series of 46 total patients experiencing painful symptomatic total knee arthroplasty deemed revisable without a past medical history of an allergic reaction to a metal implant. Index implants included nickel containing devices from a variety of name brand companies including Zimmer Biomet, Smith & Nephew^®^, and Stryker^®^. Each patient was evaluated with anterior/posterior, lateral, and individual component spot view radiographs to assess for implant malposition, patients with concern for instability underwent stress radiographs to rule out ligament instability. Experimental subjects included patients whose findings were merely discomfort, swelling, instability including fibrosis restricting range of motion, or functional dissatisfaction without significant radiographic findings and were classified as “non-radiographic” meaning that these patients were unsatisfied with their implants without a specific radiographic cause. We chose for comparison this group of patients because we hypothesized that the unexplained, non-radiographic failure could be explained by an allergic response to the implant in the nickel sensitive group. 28 patients were assigned to the nickel sensitive group according to the results of an LTT demonstrating reactive nickel sensitivity. 18 patients had no to low reactivity of the LLT and were classified as controls. Those patients who demonstrated excessive wear byproducts by radiographically-significant polyethylene wear or arthroplasty usages of greater than 2 years were excluded from comparison, as high concentrations of wear byproducts may increase the background cellular reactivity of the CD8^+^ cell line to overshadow the increase in the CD4^+^ cell line, masking metal reactivity. 7 such patients were excluded in the nickel sensitive group and 4 were excluded in the control group. Infection exclusion criteria included elevated serum erythrocyte sedimentation rate and C-reactive protein, synovial leukocyte count, synovial neutrophil percentage, presence of purulence, culture, and > 5 neutrophils per high power field × 5 fields at 400× (Parvizi et al. 2011), in addition patients with a synovial biopsy containing ≥5 neutrophils per high power field × 5 fields at 400× were excluded from the study. This excluded 2 patients in the nickel sensitive group and 1 in the control group. After these exclusions, our cohort included 32 patients total: 19 in the nickel sensitive group (7 male, 12 female, average age 65, range 44–81) and 13 in the control group (6 male, 7 female, average age 63, range 46–82).

### Pathology

Surgical specimens were biopsied from the synovial membrane of knee directly inferior to the patella at the time of surgery for intraoperative evaluation to rule out infection and for routine clinical processing. This location was chosen due to consistent access and the degree of inflammation in this area. The formalin fixed and paraffin embedded sections were evaluated on hematoxylin and eosin stains to identify the areas with highest concentration of lymphocytic infiltration. The same sections were than stained with CD4^+^ and CD8^+^ immunohistochemical stains (Ventana, Tucson, Arizona, USA), to assess the infiltration of CD4^+^ and CD8+ cells in the synovium. The histological sections were read by 2 independent investigators and scored for the number of CD4^+^ and CD8^+^ T-lymphocytes per high-power field. A ratio of CD4^+^/CD8^+^ T-cells was then calculated. CD4^+^ T-cells, indicative of delayed-type hypersensitivity, were a presumed indication of metal sensitization.

### Surgery

All surgeries were performed in the same fashion by the same surgeon in the same institution. Both arms were revised using a constrained posterior cruciate-sacrificing implant with a multi-layer zirconium nitride-coated CoCr29Mo6 base material implant designed for reduced ion and wear byproduct emission in either an allergic sensitive Columbus^®^ (*n* = 29) or EnduRo^™^ (*n* = 3) AS knee revision system (Aesculap, Tuttlingen, Germany).

### Patient follow-up

Follow-up in both cohorts after surgical revision of implant failure was 2.5 years. Assessments for length of stay and adverse events were also evaluated as were any long-term complications out to 2 years. Functional and clinical (Knee Society Clinical Rating System) scores along with range of motion evaluations were performed at each postoperative visit to compare them to baseline values obtained prior to surgery. The functional score is measured from 0 to 100 and includes the number of blocks one can walk, the ability to use stairs, and the type of walking aid used. The clinical score is also measured from 0 to 100 and measures pain, amount of flexion contracture, extension lag, total range of flexion, alignment, and antero-posterior/mediolateral stability. The differences in the pre-operative and post-operative scores were calculated. To supplement the insensitivity of the Knee Society Score, patients were also assessed by personal interview for subjective improvement after revision to see if they were happy with their result and felt it was worthwhile to do the revision.

### Statistics

Statistics were performed using SPSS. A Shapiro-Wilk normality test was used to determine the normality of the CD4^+/^CD8^+^ ratio data set. It was determined that this data was not normally distributed (*p* < .001). Differences between the ratio of CD4^+/^CD8^+^ cells were compared between groups using a nonparametric 2-tailed Mann-Whitney U test at a 95% significance level. Cell counts, functional score, clinical score, and range of motion were compared between groups using a 2-tailed t test at a 95% significance level. To determine the appropriate sample size of the study, several assumptions were made for the *t* test employed to determine significance of the endpoint outcome. First, a two-tailed distribution was used with level α = 0.05 representing 5% probability of type I error, or equivalently, 5% probability of a false positive. Next, the power was established at the 90% representing 10% probability of type II error, or equivalently, 10% probability of a false negative. A-priori statistics were determined to compute the sample size. The meaningful effect size for our proposed study was chosen to be 0.5. Taking these a-priori statistics into consideration, the size of the study was computed to be a total of 35 subjects.

## Results

**Fig. 1:**
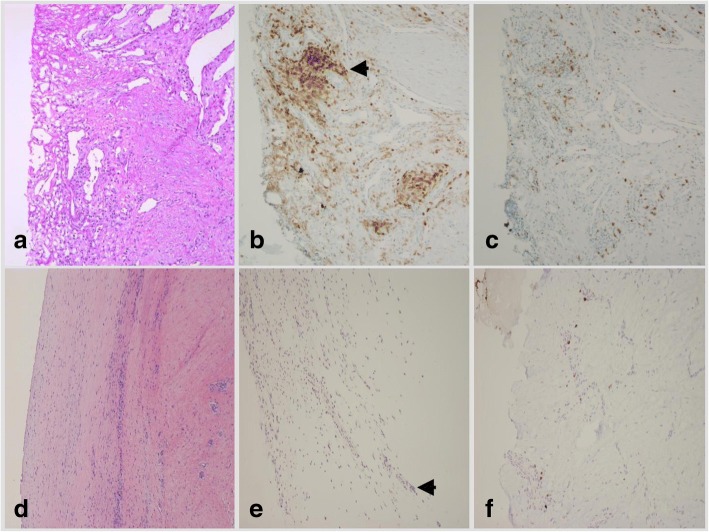
(**a**) Chronic inflammatory nidus in a nickel-sensitive synovium after total knee arthroplasty (H&E × 40). Immunohistochemical staining for CD4^+^ T-cell (**b**) and CD8^+^ T cell (**c**) markers shows a relative predominance of CD4^+^ infiltrate, consistent with a delayed-type hypersensitivity (CD4^+^ immunohistochemistry X 40). (**d**) Synovial sample from a patient in the control group (H&E × 40). Immunohistochemical staining for CD4^+^ T cell (**e**) and CD8^+^ T cell (**f**) markers shows similar levels of inflammatory infiltrate (CD8^+^ immunohistochemistry × 40). Black arrowhead denotes CD4+ staining cells

**Fig. 2:**
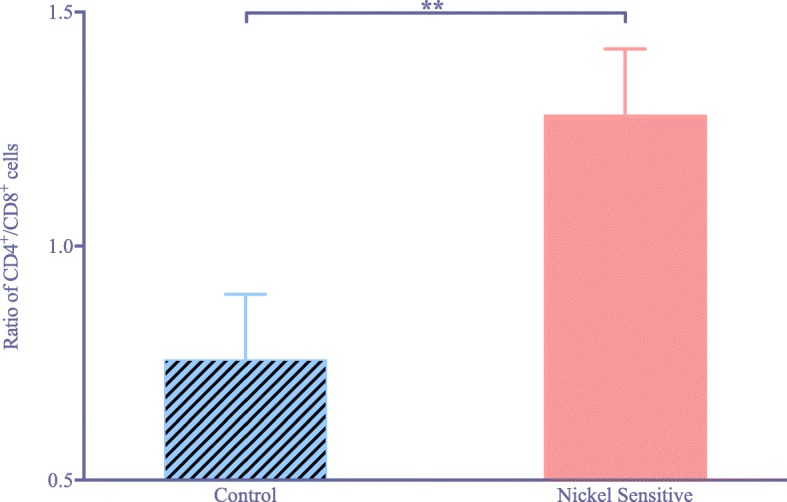
Comparison of the ratio of CD4^+^ to CD8^+^ T cells between control (*n* = 13) and nickel sensitive (*n* = 19) groups. The control average is 0.76 and the nickel sensitive average is 1.28. Error bars show standard error. **Indicates *p* = 0.009

**Fig. 3:**
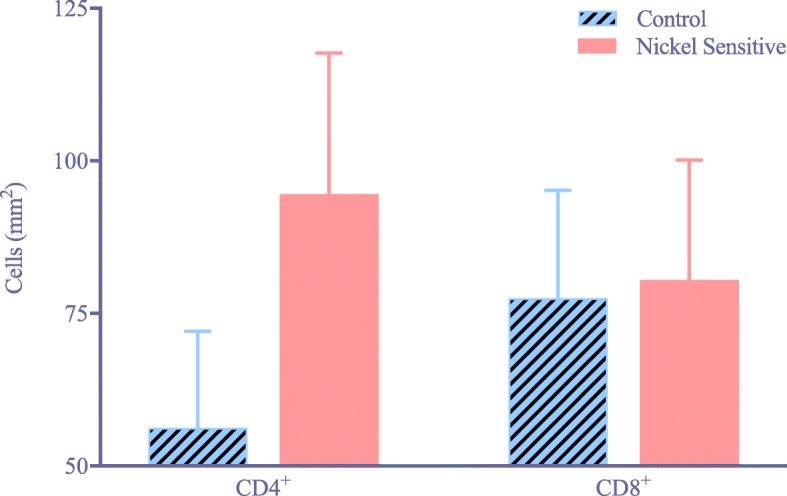
Comparison of CD4^+^ and CD8^+^ T cell counts between control (*n* = 13) and nickel sensitive (*n* = 19) groups. Control patient synovial samples show a trend of less CD4^+^ counts relative to nickel sensitive samples. Error bars show standard error

**Fig. 4:**
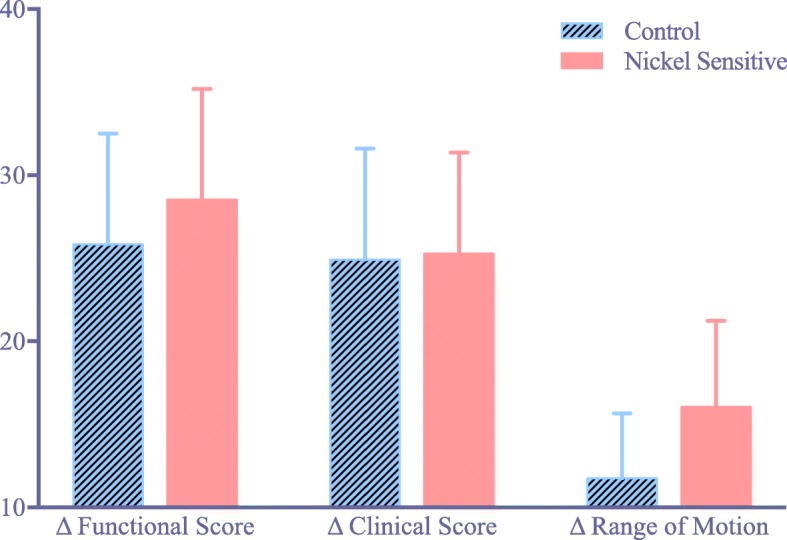
Average increase in range of motion, clinical, and functional Knee Society scores between control and nickel sensitive patients. Error bars show standard error. No significant difference in improvement of functional score, clinical score, or range of motion was found between the control (*n* = 13) and nickel sensitive (*n* = 19) groups

In the nickel sensitized group of patients, there was a significant increase in CD4^+^ reactivity compared to the CD8^+^ reactivity, while the control group showed lower amounts of inflammatory CD4^+^cells (Fig. 1). There was significant increase in the ratio of CD4^+^/ CD8^+^ lymphocyte activity between the nickel sensitive and control groups (Fig. 2). The average ratio was 1.28 in the nickel sensitive group, almost a 70% increase than that of the control average of 0.76 (*p* value = 0.009). There is a higher trending number of CD4^+^ lymphocytes in nickel sensitive patients, as shown in (Fig. 3). With greater than 2.5 year follow-up, the nickel sensitive patients, for which there was no radiographic explanation or functional aberrance to explain their chronic pain, demonstrated an average improvement of the functional knee score by over 28 points, the clinical score by 25 points, and the range of motion by 16 degrees, as shown in (Fig. 4). These functional, clinical and range of motion improvements were like those seen in the control group; there was no difference seen between the nickel sensitive and control groups. In addition to the functional knee score, clinical knee score, and range of motion results, patients were assessed for subjective improvement after revision. 14 out of 19 nickel sensitive patients (74%) noted improvement after revision. In this group, 3 patients developed stem pain for which revision to a larger stem corrected their symptoms. 1 more revision was performed for instability. Another 2 patients reported dissatisfaction with their outcomes initially without clinical signs of implant complication, although after 1 year of follow-up, 1 of these patient’s dissatisfaction was resolved. No infections occurred in either group. No chronic effusion or dermatologic manifestation of continued allergic response was seen in either group. There were no revisions in the control group.

## Discussion

The goal of this study is to examine the synovial cell counts of patients who are sensitive to nickel, as identified by the LTT. The study showed a statistically significant (*p* = 0.009) increase in the CD4^+^/CD8^+^ ratio in nickel sensitive patients by almost 70%. In addition, patients improved in function using a metal byproduct reducing implant to an equivalent level as those in the control group without a metal sensitivity. Patients with positive LTT findings prior to revision surgery of the knee may have a higher dissatisfaction with their postoperative result. While confirmation of allergic sensitization by CD4^+^/CD8^+^ ratio may provide a clue in suspected synovial samples, it is not sensitive enough to rely on without indolent LTT testing. Rather, this novel testing provides experimental evidence of the existence of metal hypersensitivity in failed TKA. Our histological results are consistent with a type IV delayed-type hypersensitivity reaction occurring in metal sensitive patients. The increased ratio of CD4^+^/CD8^+^ cells on histology was a significant finding in the nickel sensitive group. While some enhancement of CD8^+^ activity is noted in both groups, this would typically be expected as all implants exhibit some wear debris load due to years of use. This, in fact, was demonstrated by our results in this study. Notably, the most profound ratio values are found in recently implanted patients where the CD8^+^ cell line has not yet matured as a response to wear byproduct burden. Further, this series was derived from patients with a positive metal sensitivity and painful, failed total knee arthroplasty where no mechanical etiology of failure was apparent except fibrosis and loss of range of motion.

Work by numerous authors has suggested that a delayed type T-cell hypersensitivity is possible in a logical cascade of patients with failed joint replacement (Bisschop et al. 2013; Lohmann et al. 2013). In women the incidence of metal sensitization is higher because of their use of dental, jewelry and cosmetic products all of which cause increased exposure to metal (Ikarashi et al. 2002; Sicilia et al. 2008). In patients who have orthopedic implants, as compared to the normal population, the incidence of metal sensitization reaches 25% (Mihalko et al. 2012). If the implant happened to be functioning poorly, the incidence is even higher. These patients have implants which are not radiographically loose, lax, or compromised mechanically except in limited range of motion. Why did these patients fail to properly rehab from their replacement in the absence of imbalance of gap balancing, loosening, or mechanical limitation? The validation of this with histology suggests the hypothesis that allergic sensitization and allergic reaction in TKA may in fact play a role in unexplained joint pain.

In our series of patients, the incidence of nickel sensitization in poorly functioning implants was similar to those of previous studies who could not attribute symptoms to a known cause. Our percentage of 59% nickel sensitivity (19 out of 32 knees without radiographic cause for failure) compares to that of other authors (10% general, 25% implanted, and 60% poor performing implants) (Hallab et al. 2001; Jacobs and Hallab 2006; Mihalko et al. 2012; Schroer et al. 2013). This series demonstrated a satisfactory functional improvement in the metal sensitive group where no obvious clinical condition which could explain their pain was present. While not ordinarily considered an impressive outcome, this series compares favorably with many authors’ findings of patients with unexplained knee pain (Caicedo et al. 2013; Cherian et al. 2016; Elmallah et al. 2016; Hitt et al. 2015; McNabb et al. 2015; Meneghini et al. 2015; Petersen et al. 2015; Schroer et al. 2013; Siqueira et al. 2015; Watters et al. 2010; Wilke et al. 2015), particularly knee pain that cannot be explained radiographically (Mont et al. 1996). This series demonstrates that the influence of metal sensitization was apparent and was subsequently corrected by removal of the antigenic byproducts of wear.

The revision of 4 of the total nickel sensitive group (*n* = 19) represents 21% failure at 2.5 years. This rate is similar to other authors where cementless hybrid stems were used and 17% failed at 2 years compared to 32% of the cemented stems (Edwards et al. 2014). Likewise, in a different series of 84 patients fifty-year-old or younger, 27% failed for any reason at 10 years (Aggarwal et al. 2014). An incidence of 35% visible stem hypertrophy was noted in the first year. At 3-year-follow-up, no further revisions were done and half without a cessation of tip-of-stem pain. What is worrisome is the frequency of early stem hypertrophy in the long stem cementless hybrid group of patients in which 20% were still considered at risk for continued or impending failures from continued increasing modular mismatch and cortical hypertrophy. The design of implant may influence the outcome such as hybrid cementless long stems in women prone to osteopenia and modulous mismatch. While women were more prone to this, it was apparent in both male and female patients where these stems are used (solid cobalt chrome coated stems). Surgeons should counsel patients when this choice of stem is used. Furthermore surgeons need to give realistic expectations to those patients undergoing TKA revision who are sensitive to nickel, in this report only 75% of patients were satisfied with their revision and 1 in 4 required further revision surgery.

There are weaknesses and limitations to this study. As with any initial assessment into a preliminary report, we strove to get the best histological sampling possible. Inevitably, the histological sectioning of the most inflamed region of tissues (always in the patellar recess) may either over or under represent the general reaction. Inflammation is also present in byproducts of wear as well as with loosening of implants. However, it is the CD4^+^ antigenic immunologic staining which is more prominent in delayed-type hypersensitivity reactions, whereas in loose or unstable TKA, high loads of byproducts of wear are present resulting in CD8^+^ cellular hyperplasia. It is with this difference in reactivity and stain uptake that we sought to eliminate the lack of differentiation that exists on histological sections of H&E staining, which generally reveals inflammation, but does not distinguish from which cell line the inflammation originated. Although nickel sensitization, as measured by LTT, was associated with a higher CD4^+^/CD8^+^ ratio, this does not determine absolute causality. An elevated CD4^+^/CD8^+^ ratio would be expected to be seen in any Type-IV inflammatory reaction. This could be attributed to sensitivity to other metals present in implants, polyethylene insert, exposure to metals from surgical instruments and saws, or bone cement exposure.

The implant used in these revisions was exclusively that of an allergy-sensitive resistive implant. It allegedly reduces, but does not eliminate, the wear byproducts. Just as other implants attempt to reduce the immunogenic load through implants such as molybdenum, coatings, all-poly trays, and cement bed incorporation to contain exposures to raw metal surfaces, total elimination of metal byproducts has not been achieved. To give the best possible reduction in immunogenic exposure, a ceramic-coated implant was used to provide this relative reduction to best validate our study results postoperatively. While a cohort of coated versus non-coated implants might provide more evidence for allergy existence in the future, this study may provide more immediate information for the surgeon to be able to counsel patients when less obvious reasons for knee pain exists, short of an allergic sensitization. This is the first reported series of patients with known metal sensitization according to an LTT, which shows a histologically different synovium from a control, giving some credence to the fact that metal sensitivity may be a more important etiology of radiographically-unexplained implant failure than initially appreciated. In addition, use of an LTT test in high risk or self-proclaimed metal sensitive patients may be of value to consider in the future so as to document and have on hand appropriate metal wear reduced implants for use in such high-risk patients. This information can also be useful for educating insurance companies on the necessity of coated implants in patients with known metal allergies. The evaluation of a CD4^+^/CD8^+^ ratio in samples of synovium is neither sensitive nor specific enough on its own to define metal allergies in patients. LTT can be positive in a patient with a metal allergy unrelated to an implant, while an elevated CD4/CD8 ratio however is more specific for an inflammatory reaction occurring in the joint synovium. The results of this study indicate that metal sensitivity may exist and may be an important factor to consider in implant selection. Moreover, a patient’s synovial CD4^+^/CD8^+^ ratio may be a useful addition to the LTT and clinical suspicion in determining the etiology of early failed knees.

As more vendors are offering metal byproduct reduced coatings and/or alloys, which are consistent, allergic sensitization may be less of an issue in the future. While not a huge source of implant complications, metal byproduct allergy may play a larger role than previously suspected.

## Abbreviations

ALVAL: aseptic lymphocyte-dominated vasculitis-associated lesion
LLT: lymphocyte stimulation test
MOM: metal-on-metal
TKA: total knee arthroplasty
